# Reversal of Ampicillin Resistance in MRSA via Inhibition of Penicillin-Binding Protein 2a by *Acalypha wilkesiana*

**DOI:** 10.1155/2014/965348

**Published:** 2014-06-30

**Authors:** Carolina Santiago, Ee Leen Pang, Kuan-Hon Lim, Hwei-San Loh, Kang Nee Ting

**Affiliations:** Faculty of Science, University of Nottingham Malaysia Campus, Jalan Broga, 43500 Semenyih, Selangor, Malaysia

## Abstract

The inhibitory activity of a semipure fraction from the plant, *Acalypha wilkesiana* assigned as 9EA-FC-B, alone and in combination with ampicillin, was studied against methicillin-resistant *Staphylococcus aureus* (MRSA). In addition, effects of the combination treatment on PBP2a expression were investigated. Microdilution assay was used to determine the minimal inhibitory concentrations (MIC). Synergistic effects of 9EA-FC-B with ampicillin were determined using the fractional inhibitory concentration (FIC) index and kinetic growth curve assay. Western blot experiments were carried out to study the PBP2a expression in treated MRSA cultures. The results showed a synergistic effect between ampicillin and 9EA-FC-B treatment with the lowest FIC index of 0.19 (synergism ≤ 0.5). The presence of 9EA-FC-B reduced the MIC of ampicillin from 50 to 1.56 *μ*g mL^−1^. When ampicillin and 9EA-FC-B were combined at subinhibitory level, the kinetic growth curves were suppressed. The antibacterial effect of 9EA-FC-B and ampicillin was shown to be synergistic. The synergism is due the ability of 9EA-FC-B to suppress the activity of PBP2a, thus restoring the susceptibility of MRSA to ampicillin. Corilagin was postulated to be the constituent responsible for the synergistic activity showed by 9EA-FC-B.

## 1. Introduction

Infection caused by methicillin-resistance* Staphylococcus aureus* (MRSA) is a world-wide health problem. These infections are predominantly observed among immunocompromised patients in hospitals. However, in recent years, there have been an increasing number of fatalities, failed treatment, and healthcare costs. Progressive escalation of antibiotic resistance in MRSA has resulted in a limited option for treatment [1]. Thus, there is an immediate need for alternative therapies to control the spread of illness caused by MRSA.

A key strategy in combating a resistant microorganism is to suppress its resistance factor. There are at least two mechanisms that* Staphylococci* can evade beta-lactam toxicity which are by synthesizing the penicillin-binding protein 2a (PBP2a) and *β*-lactamases [2]. In normal circumstances,* Staphylococcus aureus* strains produce penicillin-binding proteins (PBPs) for synthesis of bacterial cell wall. However, when exposed to lethal concentration of beta-lactams antibiotics, resistant* S. aureus* strains produce PBP2a, which has unusually low binding affinity to beta-lactams [3]. PBP2a, in turn, replaces the function of normal PBPs (blocked by beta-lactams) in the resistant strains for cell wall biosynthesis [4]. This confers MRSA resistance to the entire beta-lactam family. Hence, suppression of PBP2a production is a promising approach to overcome MRSA's resistance. By doing so, there is a possibility to restore the susceptibility of MRSA to beta-lactam antibiotics [5].

It has been observed that extracts, fractions, or metabolites of plant origin are able to inhibit production of PBP2a when they are used in combination with current available antibiotics. In a review article [6], combinations of drugs such as beta-lactams and beta-lactamase inhibitors of plant origin have been shown to exhibit synergistic activities against antibiotic resistant microorganisms. The article also highlights plants as a source of small molecule antibiotics and synergism observed in natural products with antibiotics against growth of bacteria, fungus, and mycobacteria. Using combination treatment as a strategy to tackle resistant microorganism has also demonstrated several successes at experimental level. For an example, combination of corilagin from the plant,* Arctostaphylos uva-ursi*, with oxacillin successfully inhibited production of PBP2a in MRSA. The MIC of oxacillin and other tested beta-lactams was reduced between 100-fold and 2000-fold, strongly suggesting that combination treatment can potentially be an alternative method to combat virulence of MRSA [7].

*A. wilkesiana* is a medicinal plant which has widely been utilized for treating bacterial and fungal infections [8]. In some cases, the plant is also used to treat malaria, gastrointestinal problems [9], and potentially cancers [10, 11]. Previously, we found anti-MRSA and other antibacterial activities in the ethyl acetate and ethanol extracts of* A. wilkesiana* [12]. In continuation to our earlier findings, we have now embarked to further investigate the effects of the semipure extracts of* A. wilkesiana* in the reversal of ampicillin resistance in MRSA.

## 2. Methods

### 2.1. Plant Extraction

The plant material was collected from Broga, Selangor, Malaysia (September, 2010). Voucher sample is deposited in the herbarium of Faculty of Science, University of Nottingham Malaysia Campus, and assigned as UNMC 9. The dried plant material (3.6 kg) consisting of the whole plant was subjected to sequential extraction using* n*-hexane, followed by ethyl acetate and finally 95% ethanol [13, 14].

### 2.2. Isolation of Bioactive Fraction 9EA-FC-B

The ethyl acetate extract of* A. wilkesiana* (9EA) was fractionated by using vacuum liquid chromatography (silica gel). The solvent system used for elution was* n-*hexane (He) with increasing amount of chloroform (CHCl_3_) and CHCl_3_ with increasing amount of methanol (MeOH) [He/CHCl_3_ (1 : 1) → CHCl_3_ → CHCl_3_/MeOH (97 : 3 v/v) → CHCl_3_/MeOH (95 : 5 v/v) → CHCl_3_/MeOH (93 : 7 v/v) → CHCl_3_/MeOH (90 : 10 v/v) → CHCl_3_/MeOH (85 : 15 v/v)]. Fractions obtained were further fractionated via preparative centrifugal thin layer chromatography (silica gel) using a similar solvent system. The semipurified fractions were then tested for anti-MRSA activity.

### 2.3. Microorganism and Growth Conditions

Methicillin sensitive* S. aureus* ATCC 11632 (MSSA) was grown in tryptic soy broth (TSB) (Hi-Media, India) at 37°C for 24 h with a shaking mode of 220 rpm. Aliquot from this suspension was streaked on tryptic soy agar (TSA) (Hi-Media, India) and incubated at 37°C for another 24 h. Two to four single colonies from the TSA plate were inoculated in 10 mL of Muller Hinton broth (MHB) (Hi-Media, India) and allowed to grow at 37°C until they reached exponential stage (2 × 10^8^ CFU mL^−1^). The suspension was then used for microbroth dilution assay. MRSA ATCC 43300 was grown similarly except all the media used was supplemented with 2% sodium chloride (NaCl) (Merck, Germany), and incubation temperature was at 35°C. Bacterial stocks were kept at −80°C in TSB added with 10% (v/v) glycerol (Sigma, USA).

### 2.4. Test Samples

The crude ethyl acetate extract of* A. wilkesiana*, 9EA, and a bioactive fraction derived from it 9EA-FC-B (identified from previous experiments) were dissolved in dimethyl sulfoxide (DMSO) (Sigma, USA) at stock concentration of 100 mg mL^−1^. Further dilution was carried out using media, and the final concentration of DMSO in the media did not exceed 1%. Our earlier study has reported the lack of solvent (DMSO) effect in the test samples [14]. Antibiotics for susceptibility testing were prepared at 10 mg mL^−1^ in sterile distilled water. Tested antibiotics were ampicilin (Amresco, USA), oxacillin (Discovery Fine Chemicals, UK), and methicillin (Sigma, USA).

### 2.5. Determination of MIC

MICs of antibiotics, crude extract 9EA, and the active fraction 9EA-FC-B against MRSA and MSSA were determined via microdilution assay with a 96-well plate. Test samples were tested in a twofold serial dilution. Antibiotics were tested with concentrations ranging from 0.19 to 100 *μ*g mL^−1^ and plant extract samples from 0.09 to 12 mg mL^−1^.

MSSA and MRSA broth cultures were grown in MHB and MHB + 2% NaCl, respectively, until an exponential stage (2 × 10^8^ CFU mL^−1^) was reached (see Section 2.3). These broth cultures were diluted to correspond to final inoculums of 5 × 10^5^ CFU mL^−1^ upon inoculation into each well containing twofold serial dilutions of test sample. Media used in the assay were MHB for MSSA and MHB +2% NaCl for MRSA. In the final incubation step, plates were incubated for 24 h at 37°C for MSSA and at 35°C for MRSA. General guidelines for this experiment were obtained from Clinical and Laboratory Standards Institute 2007 [15] with recommendations adapted from several other studies [16–18].

### 2.6. Synergistic Studies

#### 2.6.1. Growth Curves Assay

MRSA was grown in a 96-well plate in the presence of following subinhibitory concentrations of 9EA-FC-B; 1/4 × MIC (0.75 mg mL^−1^), 1/8 × MIC (0.38 mg mL^−1^), and 1/16 × MIC (0.19 mg mL^−1^) in combination with subinhibitory concentrations of ampicillin ranging from 1/2 × MIC to 1/64 × MIC (25 to 0.78 *μ*g mL^−1^) under aerobic condition. Cell growth was monitored by reading optical density (OD) values at 600 nm at indicated time points for 24 h. Reading was monitored by using Varioskan Flash Multimode Reader (Thermo Scientific, USA).

#### 2.6.2. FIC Index Interpretation

FIC index for the combination treatments in synergy growth curves assay was calculated. The formula used was FIC ampicillin = MIC of ampicillin in combination/MIC ampicillin alone, FIC plant extract = MIC of plant extract in combination/MIC of plant extract alone, and FIC index = FIC ampicillin + FIC plant extract. The combination was defined as synergy if the FIC index was ≤0.5, indifference was defined >0.5 but ≤4.0, and antagonism was defined as when the FIC index was >4 [19].

### 2.7. Protein Extraction

MRSA was grown in MHB + 2% NaCl in the presence of subinhibitory concentrations of 9EA-FC-B until late exponential phase. The bacterial lysates were prepared in an extraction buffer containing Tris and EDTA, and culture supernatants were harvested via centrifugation 4340 g at 4°C for 10 min. The collected pellets were then treated with 150 mg mL^−1^ lysozyme, DNase, and protein inhibitors cocktail before being subjected to 2 h incubation at 37°C. To enhance cell disruption, 15-minute sonication in ice-bucket was done. Following 15 min of centrifugation at 13 850 g, the pellets were obtained as the insoluble protein extracts that were harvested in elution buffer containing Tris, urea, and sodium dihydrogen phosphate. Protein concentrations were measured using Pierce 660 nm protein assay.

### 2.8. SDS-PAGE and Western Blot Assay

Extracted protein (3 *μ*g mL^−1^) was stained with 4X lithium dodecyl sulphate (LDS) sample buffer and subjected to sodium dodecyl sulfate (SDS)—polyacrylamide (12%) gel electrophoresis run at 120 V. Upon completion, the gel was stained in Coomassie Blue staining solution until a clear background was obtained for scanning with GS-800 calibrated densitometer (Bio-Rad, USA). In western blot analyses, electrophoresed gels were transferred to BioTrace NT nitrocellulose transfer membrane (Pall, USA). Membranes were incubated overnight at 4°C in gelatin from cold water fish skin (blocking agent) (Sigma, USA). The production of PBP2a from MRSA was detected by probing the membranes with mouse anti-PBP2a primary antibody (Denka Seiken, Japan) and antiglyceraldehyde 3-phosphate dehydrogenase (GAPDH) (Thermo Scientific, USA) with a dilution factor of 1 : 10000. The same membranes were hybridized with anti-mouse horseradish peroxidase-linked secondary antibody (Abcam, UK) diluted to 1 : 10000 to facilitate colorimetric detection with 3,3′,5,5′-tetramethylbenzidine (TMB) substrate (Nacalai Tesque, Japan). Assay response was recorded using GS-800 calibrated densitometer (Bio-Rad, USA). Densitometric quantification of western blot images was done using Image J 1.38 programme (Windows version of NIH Image). Results were scored in percentage of expression (%) normalized to GAPDH control.

## 3. Results

### 3.1. Anti-MRSA Activity of Antibiotics and* A. wilkesiana* Extract

The MIC values of ampicillin, crude extract of* A. wilkesiana* 9EA, and fraction 9EA-FC-B for MRSA ATCC 43300 and MSSA 11632 are presented in Table 1. The MIC value of ampicillin against MRSA notably confirmed the resistance of the studied strain, while MIC value of ampicillin against MSSA revealed the susceptibility of this strain to the antibiotic. Crude extract 9EA demonstrated antibacterial activity against both the resistant and sensitive strains tested with a lower MIC value observed for MSSA. The anti-MRSA activity of fraction 9EA-FC-B was fourfold more effective than crude extract 9EA.

### 3.2. Synergistic Effects of 9EA-FC-B with Ampicillin on MRSA Growth Curves

**Figure 1:**
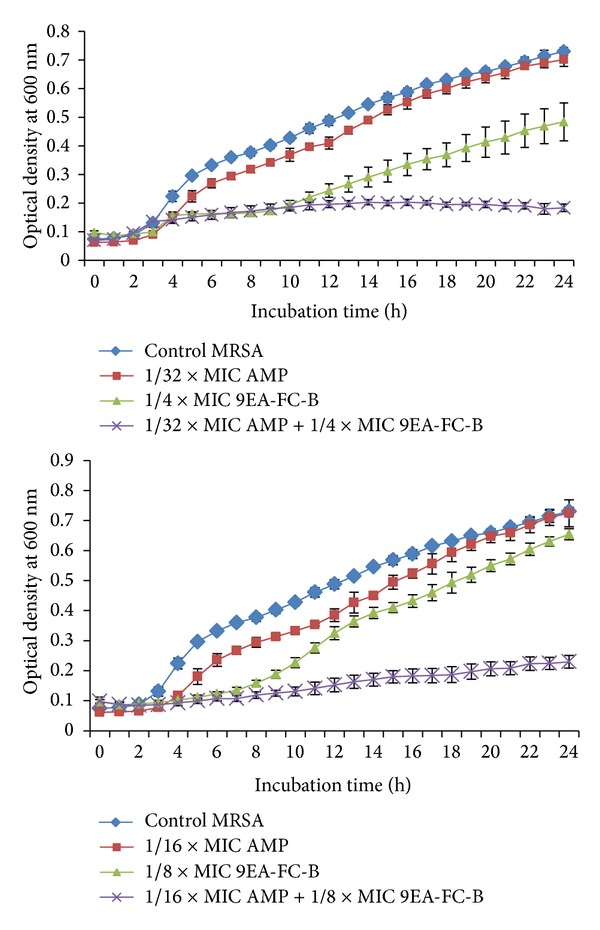
Effects of subinhibitory concentration of ampicillin alone, 9EA-FC-B alone and combination treatment on the growth of MRSA. Cell growth was measured by using OD at 600 nm at indicated time points. The curves represent triplicates of three independent experiments. Error bars show the standard deviation (AMP = ampicillin, MIC = minimum inhibitory concentrations).

The synergistic effects of 9EA-FC-B with ampicillin at subinhibitory concentrations on the growth of MRSA are shown in Figure 1. The growth inhibitory effects shown by both ampicillin and 9EA-FC-B on MRSA are concentration dependent, while the curve for the untreated MRSA culture (control) showed an exponential growth. Suppression of MRSA growth was detected when the MRSA cultures were treated with 9EA-FC-B alone at 1/4 × MIC, 1/8 × MIC and 1/16 × MIC (not shown in graph). The growth curves suggested enhanced growth inhibitory effects when MRSA was treated with ampicillin at subinhibitory concentrations in the presence of 9EA-FC-B also at subinhibitory concentrations. The inhibitory effect was observed at as low as 1/32 × MIC ampicillin in the presence of 1/4 × MIC 9EA-FC-B. Similar inhibitory effect was also achieved in the presence of 1/8 × MIC of 9EA-FC-B in combination with 1/16 × MIC ampicillin.

### 3.3. Synergistic Effects of 9EA-FC-B with Ampicillin Based on FIC Index

The FIC indices for the tested combinations are presented in Table 2. Synergistic effects were observed when 9EA-FC-B was introduced in the treatment at 1/4 × MIC, 1/8 × MIC, and 1/16 × MIC with ampicillin at subinhibitory concentrations (1/4 to 1/32 × MIC). In the presence of 1/4 × MIC 9EA-FC-B, synergistic effects were observed for the widest range of subinhibitory concentrations of ampicillin, and the range gradually decreased as the subinhibitory concentration of 9EA-FC-B was lowered.

Analysis of the FIC indices revealed the new MIC values of ampicillin in the presence of 9EA-FC-B at subinhibitory level (Table 3). 9EA-FC-B at subinhibitory concentrations has enhanced the activity of ampicillin by up to 32-fold against MRSA; for example, MIC of ampicillin alone is 50 *μ*g mL^−1^, while the MIC of ampicillin in the presence of 9EA-FC-B at 1/4 × MIC is 1.56 *μ*g mL^−1^. It is notable that the MIC values of ampicillin (for MRSA) in the presence of 1/4 × MIC 9EA-FC-B and 1/8 × MIC 9EA-FC-B are lower than the MIC value of ampicillin for MSSA, that is, 6.25 *μ*g mL^−1^.

### 3.4. Expression of PBP2a

**Figure 2:**
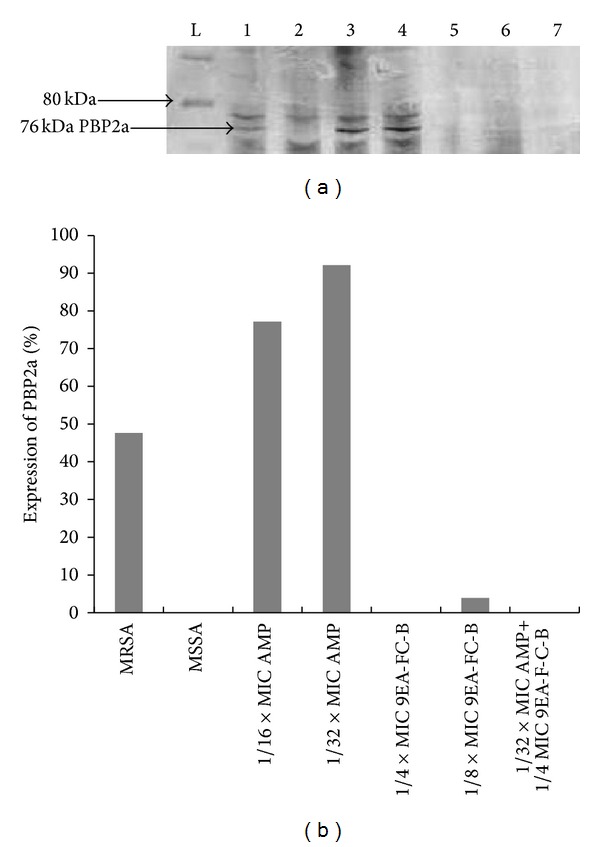
Expression of penicillin-binding protein 2a (PBP2a) of MRSA cultures grown in the presence of subinhibitory concentrations of 9EA-FC-B and ampicillin. (a) Western blot image, lane, L; molecular mass markers, 1: control MRSA, 2: control MSSA, 3: 1/16 × MIC AMP, 4: 1/32 × MIC AMP, 5: 1/4 × MIC 9EA-FC-B, 6: 1/8 × MIC 9EA-FC-B, 7: 1/32 × MIC AMP + 1/4 × MIC 9EA-FC-B. (b) Quantitative densitometric analysis of PBP2a expression of MRSA cultures grown in the presence of ampicillin alone, 9EA-FC-B alone, and in combinations, normalized to GAPDH loading control. (AMP = ampicillin; MIC = minimum inhibitory concentrations).

Expression of PBP2a was detected at 76 kDa. The percentage of PBP2a expression in tested treatments is summarized in Figure 2. GAPDH which served as an internal control was detected in all treatments (results not shown). The presence of PBP2a band was detected for MRSA cultures that were grown at subinhibitory concentrations of ampicillin (1/16 × MIC and 1/32 × MIC) in western blot experiment. Both western blot and quantitative densitometric analysis showed that these cultures have higher expression of PBP2a compared to the untreated MRSA culture (control). Inhibition of PBP2a expression was observed when MRSA cultures were exposed to subinhibitory concentrations of 9EA-FC-B, where no PBP2a band was seen at 1/4 × MIC 9EA-FC-B and only a very low expression (3.9%) was detected at 1/8 × MIC 9EA-FC-B. Likewise, the MRSA culture did not show the presence of PBP2a when grown in the presence of 1/32 × MIC ampicillin + 1/4 × MIC 9EA-FC-B.

## 4. Discussion

The scope of the study is to explore the potential of using active plant extracts or fractions to combat resistance in MRSA. For the first time, in 2006, U.S. Food and Drug Administration (FDA 2006) has approved a special green tea extract containing a proprietary mixture of phytochemicals (the active ingredient listed as Polyphenon E) [20], as a prescription drug for the topical (external) treatment of genital warts caused by the human papilloma virus (HPV). This example reinforces the efforts to study medicinal plant extracts for possible application in clinical practice. The present result established the antimicrobial activity of fraction 9EA-FC-B obtained from the ethyl acetate crude extract of* A. wilkesiana*. It also demonstrated synergism between the fraction 9EA-FC-B and ampicillin in overcoming resistance of MRSA by inhibiting production of PBP2a. In the presence of subinhibitory concentrations of 9EA-FC-B, the MIC of ampicillin was reduced by as much as 32-fold, from 50 *μ*g mL^−1^ to 1.56 *μ*g mL^−1^, indicating that MRSA became more sensitive to ampicillin when fraction 9EA-FC-B was introduced in the treatment. Based on these findings, we predict that the active constituents from fraction 9EA-FC-B may potentially be used for combating MRSA's virulence. Several researches have exploited the synergistic effects of natural products for drug development [21–23]. The most apt example that is closely related to the present study is the synergistic effects of corilagin and tellimagrandin I in combination with beta-lactam antibiotics on antibacterial activity against MRSA by inactivation of PBP2a [24].

Results of bacterial growth curve experiment indicated that combination of 9EA-FC-B with ampicillin (both agents at subinhibitory concentrations) distinctly suppressed the growth of MRSA in contrast to MRSA cultures that were treated with either 9EA-FC-B or ampicillin alone. Generally, MIC of ampicillin reduced when subinhibitory concentration of 9EA-FC-B increased in the combination treatment. From the kinetic growth curves, we were able to deduce that the antimicrobial action of the ampicillin and 9EA-FC-B combination was observed at the beginning of the exponential phase. A very minimal bacterial growth was seen with increase in incubation hours. Instead of growing rapidly during the exponential stage, the graph portrayed low growth of bacterial cells (approximately fourfold lower) with extended lag phase compared to the untreated MRSA (control). An extended lag phase was also detected for MRSA treated with 9EA-FC-B alone. Lag phase is the particular stage when bacteria equilibrate to adapt to the new environment by undergoing macromolecular repair and synthesis of cellular growth through DNA replications [25]. Hence, we deduced that a lengthy lag phase observed in our experiment is due to the inhibition of DNA replications that delays the cellular growth process. The prolonged lag phase of MRSA observed in this experiment is reminiscent of the action of fluoroquinolones that caused inhibition of DNA replication in MRSA, leading to a longer lag phase [26]. A potent antimicrobial action was also identified at the exponential phase in which bacterial cells were prevented from growing rapidly in the presence of 9EA-FC-B alone and in combination with ampicillin. This phenomenon showed probable interference in cell division which involves multiple rounds of DNA synthesis that are controlled by a variety of gene regulatory processes [27, 28]. The plausible mechanism of action of 9EA-FC-B observed at the exponential phase is therefore associated with the interruption of cell division that causes membrane derangements and failure in membrane functions.

As for interpretation of the FIC indices, lower indices indicate better synergism [19]. Based on the FIC indices obtained, eight out of the 18 combinations tested showed synergistic effects. The presence of 9EA-FC-B significantly enhanced the potency of ampicillin by up to 32-fold (MIC reduced from 50 *μ*g mL^−1^, in the absence of 9EA-FC-B, to 1.56 *μ*g mL^−1^, in the presence of 1/4 × MIC of 9EA-FC-B) for MRSA.

Ampicillin is a beta-lactam antibiotic that is designed to inhibit PBPs involved in late stage of peptidoglycan biosynthesis. Interference with peptidoglycan biosynthesis causes deformities in the bacterial cell wall and eventually leads to cell death due to high internal osmotic pressure. Nearly, all bacteria can be inhibited by interfering in mechanism of peptidoglycan synthesis [29]. Nevertheless, targeting this mechanism is no longer effective due to the production PBP2a in MRSA. The blocking of normal PBPs by beta-lactams did not exert effects on peptidoglycan or cell wall synthesis, because PBP2a replaces their function and ensures normal formation of cell wall in presence of lethal concentration of beta-lactam drugs [30]. However, in this study, we experimentally demonstrated restoration of ampicillin's antimicrobial activity by the addition of 9EA-FC-B at subinhibitory level.

The synergistic activity observed between 9EA-FC-B and ampicillin against MRSA was shown to be associated with inhibition of PBP2a. PBP2a is an inducible protein that regulates methicillin resistance. Its expression is heterogeneous in nature amidst level of resistance differing to the beta-lactam being used [31]. The* mecA* gene complex which encodes for this protein encompasses the regulatory genes,* mecI* and* mecR*. Interaction of beta-lactam antibiotics with these regulatory genes eventually allows expression of* mecA* in terms of production of PBP2a [32–34]. As such, the occurrence of intense PBP2a bands in MRSA cultures grown in low concentrations of ampicillin compared to the control culture in western blot experiment suggested the induction of* mecA* gene transcription. In contrast, no PBP2a bands were detected in MRSA cultures that were exposed to 1/4 × MIC of 9EA-FB-C, while PBP2a is only very mildly expressed when the concentration of 9EA-FC-B was lowered to 1/8 × MIC. This suggests that 9EA-FC-B can either inhibit the production of PBP2a or directly inactivate PBP2a. This suggestion is in agreement with the observation that 9EA-FC-B enhanced the ampicillin susceptibility of MRSA. In 2004, Shiota et al. [24] reported the antibacterial effect of corilagin and tellimagrandin I (isolated from* Arctostaphylos uva-ursi* and* Rosa canina*, resp.) against MRSA. The two polyphenolic compounds had exceptionally reduced MICs of beta-lactams for MRSA via inactivation of PBP2a. We believe that corilagin (a tannin) is also present in the test fraction 9EA-FC-B, since corilagin was previously isolated and identified by our colleagues from the same source of plant materials [35]. This is further supported by phytochemical testing that showed the presence of tannins in 9EA-FC-B (data not shown). Although Shimizu et al. [7] previously reported that corilagin enhanced antibacterial activity of various beta-lactams by 100- to 2000-fold against MRSA, 9EA-FC-B was only shown to enhance the activity of ampicillin by up to 32-fold in the present study. The stark difference in antibacterial enhancements can readily be rationalized by the fact that 9EA-FC-B is a semipure fraction that possibly contains only a small amount of corilagin in addition to many other secondary metabolites that were assumed to have negligible effect on the antibacterial activity observed.

## 5. Conclusion

From the results reported in this study, we found that the antibacterial effect of the fraction 9EA-FC-B obtained from* A. wilkesiana* and ampicillin is synergistic. The synergism is due the ability of 9EA-FC-B to suppress the production of PBP2a or directly inactivate it, leading to the restoration of the susceptibility of MRSA to ampicillin.

## Figures and Tables

**Table 1:** MIC values of ampicillin, crude extract 9EA, and fraction 9EA-FC-B against MRSA and MSSA.

Strain	MIC		
	Ampicillin (*μ*g mL^−1^)	9EA (mg mL^−1^)	9EA-FC-B (mg mL^−1^)
MRSA	50	12	3
MSSA	6.25	6	3

Values represent triplicates of three independent experiments.

**Table 2:** FIC indices of some combinations of ampicillin and 9EA-FC-B for MRSA.

Ampicillin (*μ*g mL^−1^)	9EA-FC-B (mg mL^−1^)		
	1/4 × MIC (0.75)	1/8 × MIC (0.38)	1/16 × MIC (0.19)
1/2 × MIC (25)	0.75	0.65	0.56
1/4 × MIC (12.5)	0.43	0.36	0.31
1/8 × MIC (6.25)	0.38	0.25	—
1/16 × MIC (3.125)	0.31	0.19	—
1/32 × MIC (1.563)	0.28	—	—
1/64 × MIC (0.781)	—	—	—

Values represent triplicates of three independent experiments. Index interpretation: ≤0.5 = synergy, >0.5 but ≤4.0 = indifference, and >4 = antagonism. (MIC = minimum inhibitory concentrations; — = no activity.)

**Table 3:** MIC values of ampicillin in combination with subinhibitory concentrations of 9EA-FC-B for MRSA.

Treatment	MIC (*μ*g mL^−1^) of ampicillin
Ampicillin alone	50
With 9EA-FC-B at 1/4 × MIC	1.56
With 9EA-FC-B at 1/8 × MIC	3.13
With 9EA-FC-B at 1/16 × MIC	12.5

Values represent triplicates of three independent experiments (MIC = minimum inhibitory concentrations).
